# The Necessity of Scientific Dental Research Work

**Published:** 1914-01-15

**Authors:** Arthur R. Melendy

**Affiliations:** Knoxville


### THE NECESSITY OF SCIENTIFIC DENTAL RESEARCH WORK.

BY ARTHUR R. MELENDY, KNOXVILLE.

Read before the Tennessee Dental Association, June 5, 1913.

The title of my paper does not fully indicate the subject or subjects which I desire to present for your consideration. I shall try to be brief so that I trust you may not feel that you have been imposed upon if I enlarge somewhat upon the title as contained in our program.

As a profession we have made wonderful progress during the past decade. I shall not take time to mention even a small part of what has been accomplished; step by step we have advanced upward until we are recognized in every intelligent community as one of the learned professions. That we have not kept fully abreast of the times as specialists cannot be truthfully said of us, but we must realize that much as we have accomplished, more and greater things are demanded of us. If it be true that Rheumatism, Diphtheria, Scarlet Fever, Measles, Epidemic Meningitis and a host of other diseases find their entrance to the body through the portals of an unhealthy mouth; if it be true that Dental disease is more prevalent than any other in the human family and more prolific of evil directly and indirectly; if it be true that the greater part of the infectious diseases that attack the human family have their origin in the mouth and that these diseases are spread abroad by the unkept and filthy mouths of children and adults with whom our own well kept children are forced to come into contact in our public schools, in public places of entertainment, on street cars and every where they may go; if it be true, as Dr. Brophy claims, that it is a matter of record that, save only in woman, the mouth is more subject to tumors than any other part of the body, and these tumors have as an exciting cause in a very large percentage of the cases where they appear from diseased teeth; it is high time we were securing more exact knowledge of the causes of diseases of the teeth.

I think we may eliminate the if, for we know that every suggestion which I have made above is true, yea, that it is far less than the whole truth, and by reason of these already well recognized facts the Dental profession is face to face with a stupendous responsibility; for not until we, as oral specialists, can prevent or control the septic conditions so prevalent in the mouths of the human family may the Medical profession hope for any definite progress in the science of prevention, and we all know that preventive medicine, so-called, is uppermost in the thought of the Medical profession today.

Dr. Chas. Mayo in his paper read before the recent meeting in Chicago, January 31st, 1913, said, "It is evident that the next great step in medical progress in the line of preventive medicine should be made by the dentists. The question is, Will they do it ?" and Dr. Wm. Hunter of London in a paper on oral sepsis read before the faculty of McGill University of Montreal said, "My object is to emphasize the great fact that it is not the absence of teeth but the presence of sepsis; that it is not the dental defects but the septic effects; that it is not defective mastication but effective sepsis associated with such dental defects that are responsible for the ill health associated with "bad" mouths. This matter of oral sepsis is, therefore, of urgent importance in relation to the whole multifarious and wide spread group of affections, medical, surgical and dental, caused by the actual presence or toxic action of pyogenic organisms. There can be no doubt that the future of oral pathology and treatment, and therefore of practical dentistry, depends upon the extent to which those who occupy themselves with those subjects are trained in the principals and impregnated with the fundamental truth of oral sepsis and antisepsis."

These brief extracts from two such distinguished men serve to show us the trend of sentiment among the most advanced thinkers of the Medical profession, and bring us to a consideration of our preparedness to meet the conditions and demands of the present age, a condition which we have brought upon ourselves by our very progressiveness. Are we able to measure up to the standards that seems to be necessary, the standards which we have established? I turn to the colleges and they say yes. We are graduating each year better equipped men than ever before, we are giving them more and requiring more of them. The State Examining boards will tell you that they are raising their standards for license each year, and every practitioner of ten years experience will tell you that a discerning public is demanding and is willing to pay for better service today than ever before. Yes, all of this is true and we are gratified that it is so, but that is not the point. There is no doubt in the minds of any of us about the colleges teaching in the most forcible manner the fundamental truths of Dentistry so far as they are known. There is no doubt about the examining boarde raising their standards, there is not the slightest doubt about the better class of Dentists in every community and in the whole country, striving each year to become more proficient and faithful in the discharge of their professional duties.

All of this and more has been steadily going forward for many years, and I am proud of the progress that we have made, proud of the scientific data that has been established. But my brothers, we are face to face today with many problems for which we do not know any definite scientific treatment, and it is these questions that we don't know that are becoming so insistant and demanding an authoritative solution at our hands. We must have more definite scientific knowledge about the cause and treatment of Pyorrhea Alveoloris; we must know more about the best means for preventing decay of the teeth; we must know more about the etiology and cure of erosion; of enamel atrophy; of how to bring about immunity to systemic infection from local foci; we need to have a technic that will produce less error when fusing gold or other metals; we need more perfect amalgams; a better substitute for platinum; more life-like and durable filling materials. These and many other questions can only be solved by competent trained men who shall devote all their time to research work, and this can not be done with out a considerable fund to be used solely for this purpose.

Not withstanding the fact that all the great epoch making advances in the medical science have been the result of definite laboratory research, the dental profession has no adequate facilities for research work of any kind. What has been done up to this time has been almost wholly restricted to the efforts of individual investigators, and we are all agreed that the general practitioner has not the time, the qualifications or facilities for doing this work successfully. Hence I say we are face to face today with the necessity for more adequate research work if we would retain the respect of other scientific bodies or meet the demands of suffering humanity.

Within the past week Governor Cox has appointed Dr. Homer C. Brown of Columbus upon the Ohio State board of health. The announcement came as a surprise to the medical world and is heralded in the newspapers as the first time the Dental profession has been so recognized. Dr. E. E. McCampbell, Executive secretary of the Ohio State board of health in commenting on the appointment, complimented Gov. Cox for making it and declared it to be another proof of the Governors intelligent interest in the field of public health work. This is the latest evidence which I shall present in my effort to impress upon you the exact situation of the Dental profession today. And just here let me say that those of us occupying modest places in the profession and who constitute the great majority in numbers may not hide behind the statement that these great issues must be met by our great leaders and teachers. It is not the President of a Railroad System, nor even the train dispatcher, on whom alone the safety or the wrecking of a train hangs. A right or wrong action on the part of any one of a thousand other persons may save or wreck that train. So each one of us may be sure of having his part and responsibility in providing more adequate facilities for research work. Our clientle and the community in which we serve demand that we shall not shirk this great responsibility, and I am glad to be able to tell you that the National Dental Association at the Washington meeting last September authorized a Scientific Foundation Fund for the purpose of devising ways and means for meeting this special need. Dr. Weston A. Price of Cleveland, Ohio, now President of the Ohio State Dental Society, is Chairman of that Committee, and has entered with great enthusiasm upon the work. After careful consideration it seemed evident that until Dentists should inaugurate this work and manifest some "Esprit de corps" in its interest that we could not expect wealthy philanthropists to come to our assistance. Then too it is important that the Dental profession shall not only institute but indorse morally and financially this work in order that it may be under the control of the profession, and that the Dental profession may be given full credit for such discoveries and assistance to humanity as may come. Hence, the plan adopted is to provide an immediate annual fund equal to at least $1.00 from each member of the Dental profession to be paid annually for five years. To insure this we should have an average of $5.00 per year for five years from each member of all Dental Societies to make up for the many who can not be reached or who will not be interested. This plan places the opportunity to have a part in this work within the reach of every one which should unite the whole profession in a work of common professional and humanitarian interest. Cleveland, Cincinnati, Chicago and several other places have already pledged a considerable sum and the committee are desirous of having a sufficient amount pledged by the time of the next annual meeting of the National Dental Association in Kansas City, July 8th, to insure the immediate commencement of this work.

The free use of several large well equipped pathological and one matalurgical and physical laboratory has already been secured. Also options on some of the best research men in this country and in Europe. Consequently every thing seems propitious for the early launching of actual research work and I am wondering what part Tennessee will assume. This is not, Mr. President, a question for action on the part of this society as a body, but it is an individual matter with each member, and if the whole profession shall stand together it will only be a few years till we shall have endowed chairs for research work in many of our colleges, and the benevolent rich will leave large bequests for Dental research. Shall we claim our own?

